# Supplementary material for: Direct observation of spin-layer locking by local Rashba effect in monolayer semiconducting PtSe2 film
Source: Nat Commun. 2017 Jan 31;8:14216. doi: 10.1038/ncomms14216 (PMC5290424; doi:10.1038/ncomms14216)
Supplement: Supplementary Information — Supplementary Figures, Supplementary Notes and Supplementary References. [file ncomms14216-s1.pdf]

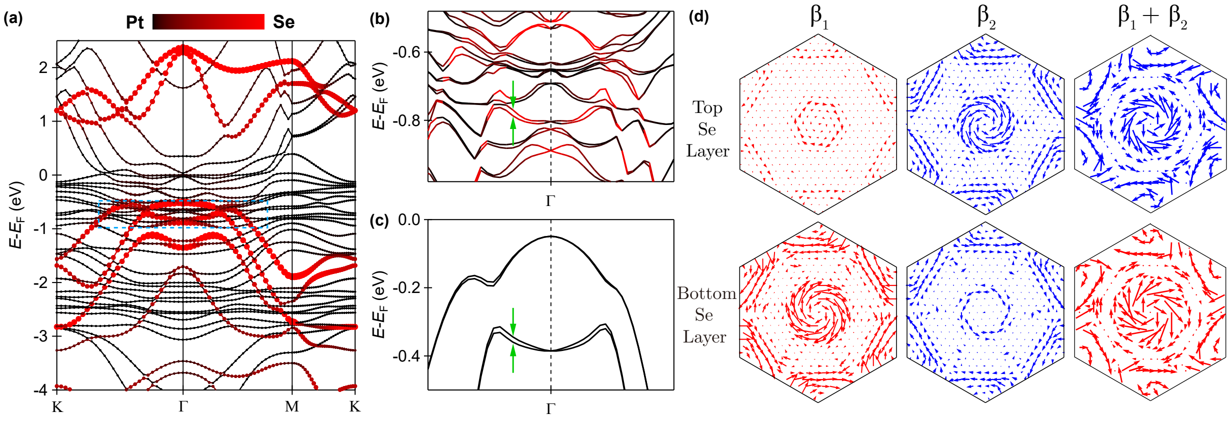

SUPPLEMENTARY FIGURE 1: **Band structure and spin texture of monolayer $PtSe_2$ in the presence of Pt(111) substrate and an external electric field.** **(a)** The calculated band structure of monolayer $PtSe_2$ on a Pt(111) substrate. The color scale indicates the contribution from the Pt or Se and the size of the dots represents the weight. **(b)** Zoom in of the blue box in **a**, the energy bands show a very small splitting due to the substrate. **(c)** the band structure of monolayer $PtSe_2$ in an external electric field with strength of 0.1 V·$Å^{-1}$. The energy bands also show a splitting with a similar gap size as **b**. **(d)** Se-layer- and sub-band-resolved spin texture for β band. The two sub-bands show opposite layer dependence. For β_1 band, the bottom Se layer is dominant for spin polarization, and for β_2 band, instead, the top Se layer is dominant. The sum of the spin polarization in the two sub-bands for top and bottom layer is shown on the right.

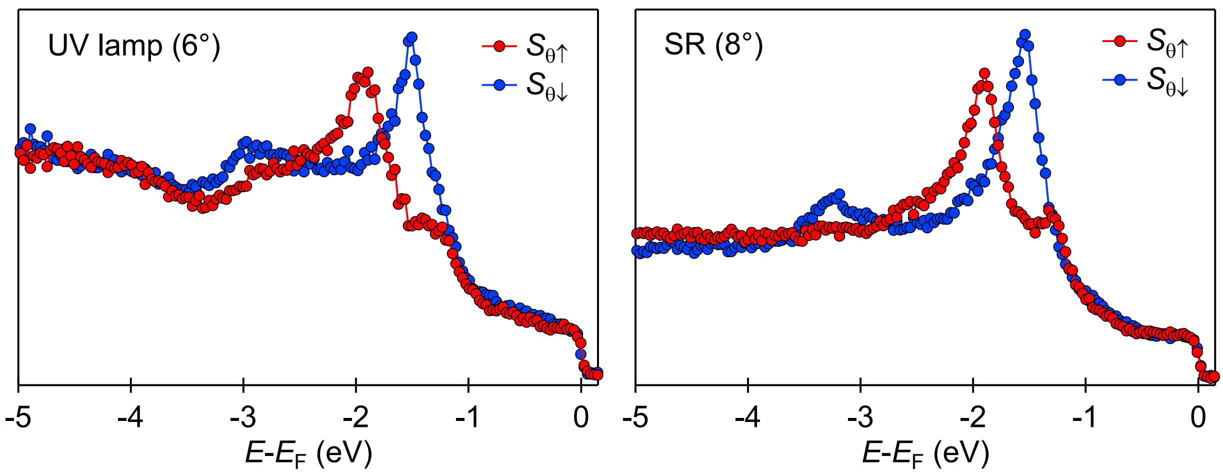

SUPPLEMENTARY FIGURE 2: **Spin-resolved energy distribution curves (EDCs) under different photon polarization.** The left panel shows the spin-resolved EDC at 6° emission angle along Γ-M direction by unpolarized UV lamp. The right panel shows the spin-resolved EDC at 8° emission angle by linear polarized synchrotron radiation. The similar results indicate that the spin polarization is not related to the polarization of light.

Supplementary Notes

SUPPLEMENTARY NOTE 1. EFFECT OF THE Pt(111) SUBSTRATE ON THE ELECTRONIC AND SPIN STRUCTURE OF MONOLAYER $PtSe_2$

Supplementary Figure 1(a) shows the calculated band structure of monolayer $PtSe_2$ on Pt(111) substrate, using an experimental value for the separation of ~ 4.5 Å between the $PtSe_2$ and the substrate [1]. To extract the energy bands of monolayer $PtSe_2$, we project these bands onto Se atoms and show them as red dots. The projected band structure still retains the band structure of free-standing $PtSe_2$ (Fig. 1(b) in main text), suggesting that the hybridization between Pt(111) and the sample is negligible. More detailed analysis (Supplementary Figure 1(b)) shows a small spin splitting of ~ 15 meV in the β band of $PtSe_2$. Therefore the effect of the substrate is mainly to induce a charge transfer and an electric field by a potential gradient. We further perform the calculation by applying a small external electric field on monolayer $PtSe_2$ to simulate the impact from substrate. Under an applied electric field of 0.1 V·Å^{-1}, the β band opens a SOC gap (Supplementary Figure 1(c)) with a similar value to that induced by the substrate, hence such an electric field could mimic the effect from substrates. Under this condition, we analyze the spin texture following the same procedure in the main text. We find that spin textures in each layer are almost identical between the case of the free-standing film and that with an external electric field. If we sum over the spin polarization over two spin split bands (e.g. β_1 and β_2 bands), two layers possess opposite spin textures (Supplementary Figure 1(d)). Thus, it is only possible for us to observe a spin texture if our spin ARPES experiment can resolve the difference between the top and bottom Se layers (the R-2 effect), but no spin texture will appear if we sum over two layers. Therefore, although the electric field from the substrate may break the global inversion symmetry and enhance the spin polarization slightly, the dominant mechanism behind the helical spin textures observed is the R-2 effect.

SUPPLEMENTARY NOTE 2. PHOTON-POLARIZATION DEPENDENCE OF SPIN POLARIZATIONS

In the spin-ARPES measurements, we use two kinds of light sources: unpolarized UV lamp at 21.2 eV and linear polarized synchrotron radiation, and obtain similar results (see Supplementary Figure 2). This confirms that the spin polarizations measured in our sample are not dependent on the photon polarization. Thus it is an intrinsic phenomenon of monolayer $PtSe_2$. Since the unpolarized light from UV lamp also gives finite spin polarization, we can rule out the possibility that the observed spin polarizations are caused through manipulating the electron spins by polarized photon.

SUPPLEMENTARY NOTE 3. ATOMIC ORBITAL NATURES OF THE BANDS

The aim of this section is to provide an analysis of the atomic orbital natures of the conduction and valence bands near the Fermi energy shown in Fig. 5(b). We focus on the p-orbitals of Se atoms for the reasons discussed in the main text. Let us label the p-orbitals of Se atoms by $|\tau, \alpha, s\rangle$, where $\tau = t, b$ is for top and bottom layers, α for $p_{x,y,z}$ orbitals and $s = \uparrow, \downarrow$ for spin. We may first consider the strong anisotropy between in-plane and out-of-plane directions, leading to the splitting between the states of p_z orbital and $p_{x,y}$ orbitals. Next we consider the bonding and anti-bonding states of Se p orbitals and the basis are changed to

$$|\eta = \pm, \alpha, s\rangle = \frac{1}{\sqrt{2}}(|\tau = t, \alpha, s\rangle \pm |\tau = b, \alpha, s\rangle), \tag{1}$$

and the hybridization of the states between top and bottom layers leads to a splitting between the bonding ($\eta = +$) and anti-bonding states ($\eta = -$), as shown by the second step in Fig. 5(b). Due to C_3 rotation symmetry, it is more convenient to make a linear combination of p_x and p_y orbitals as

$$|\eta, p_+, s\rangle = -\frac{1}{\sqrt{2}}(|\eta, p_x, s\rangle + i|\eta, p_y, s\rangle), \tag{2}$$

$$|\eta, p_-, s\rangle = \frac{1}{\sqrt{2}}(|\eta, p_x, s\rangle - i|\eta, p_y, s\rangle), \tag{3}$$

which are the eigen states of C_3 rotation. $|\eta, p_+, s\rangle$ with the z direction orbital angular momentum $L_z = +1$ can be related to $|\eta, p_-, s\rangle$ with $L_z = -1$ by time reversal symmetry,

and thus they must be degenerate at the Γ point. By including spin-orbit coupling, we need to consider the total angular momentum $\mathbf{J} = \mathbf{L} + \mathbf{S}$. As a result, the eigen-states at the Γ point can be written as

$$|\eta, J = \frac{3}{2}, J_z = \frac{3}{2}\rangle = |\eta, p_+, \uparrow\rangle \tag{4}$$

$$|\eta, J = \frac{3}{2}, J_z = -\frac{3}{2}\rangle = |\eta, p_-, \downarrow\rangle \tag{5}$$

$$|\eta, J = \frac{3}{2}, J_z = \frac{1}{2}\rangle = u_1|\eta, p_+, \downarrow\rangle + v_1|\eta, p_z, \uparrow\rangle \tag{6}$$

$$|\eta, J = \frac{3}{2}, J_z = -\frac{1}{2}\rangle = -u_1^*|\eta, p_-, \uparrow\rangle - v_1^*|\eta, p_z, \downarrow\rangle \tag{7}$$

$$|\eta, J = \frac{1}{2}, J_z = \frac{1}{2}\rangle = u_2|\eta, p_+, \downarrow\rangle + v_2|\eta, p_z, \uparrow\rangle \tag{8}$$

$$|\eta, J = \frac{1}{2}, J_z = -\frac{1}{2}\rangle = -u_2^*|\eta, p_-, \uparrow\rangle - v_2^*|\eta, p_z, \downarrow\rangle \tag{9}$$

where $u_{1,2}$ and $v_{1,2}$ are material dependent parameters. $|\eta, J = \frac{3}{2}, J_z = -\frac{3}{2}\rangle$, $|\eta, J = \frac{3}{2}, J_z = -\frac{1}{2}\rangle$ and $|\eta, J = \frac{1}{2}, J_z = -\frac{1}{2}\rangle$ can be related to $|\eta, J = \frac{3}{2}, J_z = \frac{3}{2}\rangle$, $|\eta, J = \frac{3}{2}, J_z = \frac{1}{2}\rangle$ and $|\eta, J = \frac{1}{2}, J_z = \frac{1}{2}\rangle$ by time reversal symmetry, respectively. From Fig. 5(b) in the main text, one can see that α, β and γ bands are given by $|\eta = +, \frac{3}{2}, \pm\frac{3}{2}\rangle$, $|\eta = +, \frac{3}{2}, \pm\frac{1}{2}\rangle$ and $|\eta = -, \frac{1}{2}, \pm\frac{1}{2}\rangle$. δ band comes from d_{z^2} orbital.

Next we focus on the effective Hamiltonian for the α and β bands around the Γ point based on the symmetry properties of the system. Instead of considering the bonding and anti-bonding basis (labeled by η), it is more convenient to work on the basis of two layers (labeled by τ). Let us first consider the β band with $\frac{1}{2}$ z-direction angular momentum on the top layer, the basis of which can be labeled by $|\tau = t, \frac{3}{2}, \pm\frac{1}{2}\rangle$. The corresponding effective Hamiltonian should possess three-fold rotation symmetry (C_3), the x-direction mirror symmetry (m_x) and time reversal (T). Under the basis $|\tau = t, \frac{3}{2}, \pm\frac{3}{2}\rangle$, these symmetry operators can be given by $C_3 = exp(i\pi\hat{s}_z/3)$, $m_x = i\hat{s}_x$ and $T = i\hat{s}K$ where $\hat{s}$ is the Pauli matrix and K is complex conjugate. To be invariant under these symmetry operators, the Hamiltonian takes the form

$$H_{1/2,t} = A_1 + A_2k^2 + A_3(\hat{s}_xk_y - \hat{s}_yk_x) \tag{10}$$

The effective Hamiltonian on the bottom layer can be related to that on the top layer by inversion symmetry, which reverses the momentum k, but keep the angular momentum. Thus, the effective Hamiltonian on the bottom layer is given by

$$H_{1/2,b} = A_1 + A_2k^2 - A_3(\hat{s}_xk_y - \hat{s}_yk_x). \tag{11}$$

The coupling between two layers can be described by angular-momentum-conversed hopping terms and thus the full Hamiltonian

$$H_{full} = A_1 + A_2 k^2 + A_3(\hat{s}_x k_y - \hat{s}_y k_x)\hat{\sigma}_z + t\hat{\sigma}_x, \tag{12}$$

where t is the hopping parameter and $\hat{\sigma}$ is the Pauli matrix for two layers. To evaluate spin polarization on each layer, we need to project out the spin operators on the basis $|\tau, \frac{3}{2}, \pm\frac{1}{2}\rangle$, which is given by

$$\langle \tau, 3/2, J_z|S_x|\tau, 3/2, J_z'\rangle = -v_1^2(\hat{s}_x)_{J_z J_z'} \tag{13}$$

$$\langle \tau, 3/2, J_z|S_y|\tau, 3/2, J_z'\rangle = -v_1^2(\hat{s}_y)_{J_z J_z'} \tag{14}$$

$$\langle \tau, 3/2, J_z|S_z|\tau, 3/2, J_z'\rangle = (v_1^2 - u_1^2)(\hat{s}_z)_{J_z J_z'}. \tag{15}$$

Here we have used the fact that $|p_+\rangle$ and $|p_-\rangle$ are orthogonal to each other. The the in-plane components of spin operator are proportional to $\hat{s}$. One can easily show that Rashba-type of spin polarization exists in one layer for the Hamiltonian (12). The whole derivation here can also be applied to the γ band.

For the α band with $\frac{3}{2}$ z-direction angular momentum, one can also obtain the effective Hamiltonian in a similar manner. However, on the basis $|\tau, \frac{3}{2}, \pm\frac{3}{2}\rangle$, one can see that the spin operators are

$$\langle \tau, 3/2, J_z|S_x|\tau, 3/2, J_z'\rangle = 0 \tag{16}$$

$$\langle \tau, 3/2, J_z|S_y|\tau, 3/2, J_z'\rangle = 0 \tag{17}$$

$$\langle \tau, 3/2, J_z|S_z|\tau, 3/2, J_z'\rangle = (\hat{s}_z)_{J_z J_z'}. \tag{18}$$

Thus, in-plane spin components vanishes for the effective model at lowest order terms and one has to go to higher order term for spin operator, which will depend on the momentum. This explains why spin polarization is much smaller for the α bands compared to that for the β and γ bands.

Supplementary References

[1] Wang, Y. *et al.* Monolayer PtSe2, a new semiconducting transition-metal-dichalcogenide, epitaxially grown by direct selemnization of Pt. *Nano Lett.* **15**, 4013-4018 (2015).
